# Supplementary material for: Ab initio mechanism revealing for tricalcium silicate dissolution
Source: Nat Commun. 2022 Mar 10;13:1253. doi: 10.1038/s41467-022-28932-2 (PMC8913775; doi:10.1038/s41467-022-28932-2)
Supplement: Supplementary file 1 — Supplementary information [file 41467_2022_28932_MOESM1_ESM.pdf]

Supplementary Information

Ab initio mechanism revealing for tricalcium silicate dissolution

Z. Li et al.

Supplementary Methods

DFT-based Geometry optimization method. The geometry optimization of the Ca_3SiO_5 bulk crystal and (111) surface slab were calculated within density-functional theory (DFT), implemented in the Vienna Ab Initio Simulation Package (VASP) code[1, 2]. The exchange-correlation potential was approximated within the generalized gradient approximation (GGA) using the Perdew-Burke-Ernzerhof (PBE) functional[3] due to the outstanding agreement between experimental and theoretical lattice constants[4] and the good suitability for calculation of calcium silicate species[5, 6]. The valence electrons of $3s^23p^64s^2$, $3s^23p^2$, $2s^22p^4$, and $1s^1$ were considered for Ca, Si, O and H, respectively. Iterative solutions of the Kohn−Sham equations were expanded in a plane-wave basis set defined by a kinetic energy cutoff of 600 eV [7]. The global convergence criterion of energy for the electronic self-consistent loops was set as 10^{-5} eV. The bulk unit cell was optimized with the tolerance of 10^{-3} eV Å^{-1} for the ionic relaxation[6, 8]. Monkhorst-Pack[9] scheme was used for the k-point sampling with a grid of 3 × 5 × 4 in the first Brillouin zone. surfaces were maintained neutral with integer numbers of basis to preclude the polarizing electric field[10, 11]. For surface relaxation, the vacuum was set as 20 Å with a dipole correction along the z direction was applied. The two uppermost layers of atoms were completely relaxed while the rest were fixed[12]. The lattice constants of slab models were fixed[13]. No symmetry was forced on both sides of slabs. The optimization threshold was 0.02 eV Å^{-1} for ionic relaxations and the k-points mesh is 2 × 2 × 1.

Supplementary Table 1. The coordination environments of the Ca ion on other Ca_3SiO_5 surfaces.

Miller index	Ca coordination environment
100	three-, four- and six- coordinated
010	four-,five- and six- coordinated
001	five-coordinated
110	four-coordinated
101	four-,five-, six- and seven- coordinated
011	four- and five- coordinated
111	three- and five- coordinated

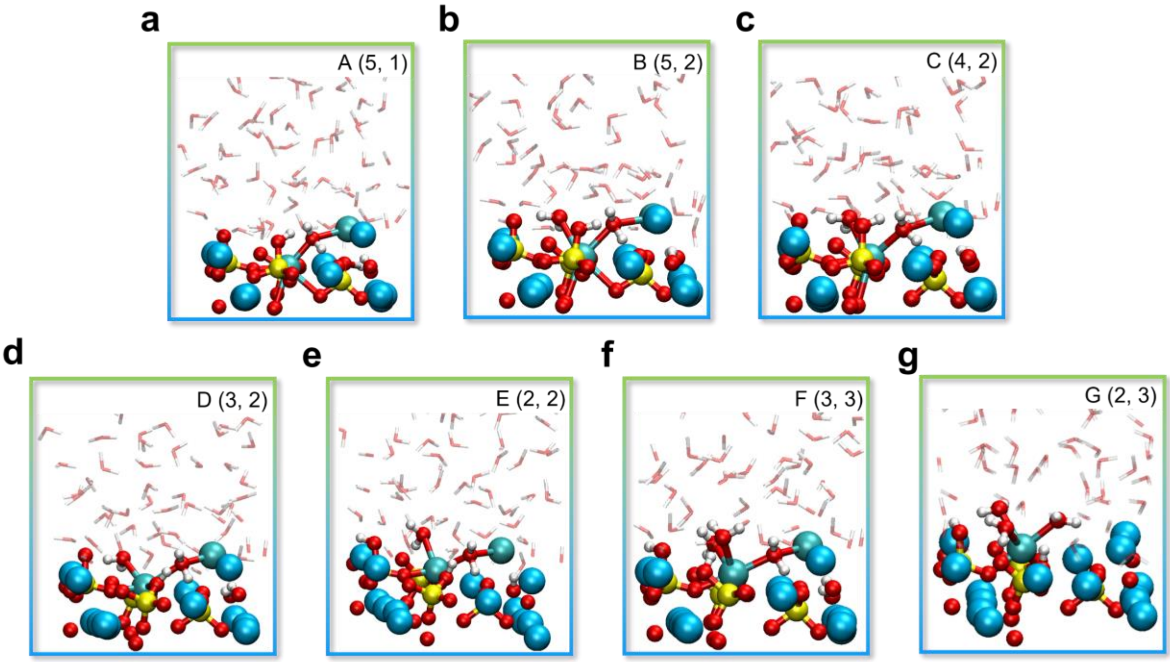

Supplementary Figure 1. The all-atom configurations of the free energy minimum states on the FES for dissolution of Ca_β with CN(Ca-O_s) from 5 to 2. **a-g** The configurations of the states from A to G. The state number and the corresponding coordinates on the FES are at the upper right. The yellow, blue, cyan, red and white spheres are indicted to the silicon, calcium with no bias potential, calcium with bias potential, oxygen and hydrogen ions, respectively. The calcium with no bias potential but connecting with Ca_β through hydroxyl group is also shown in cyan. For simplicity, the solute is shown in the transparent stick type.

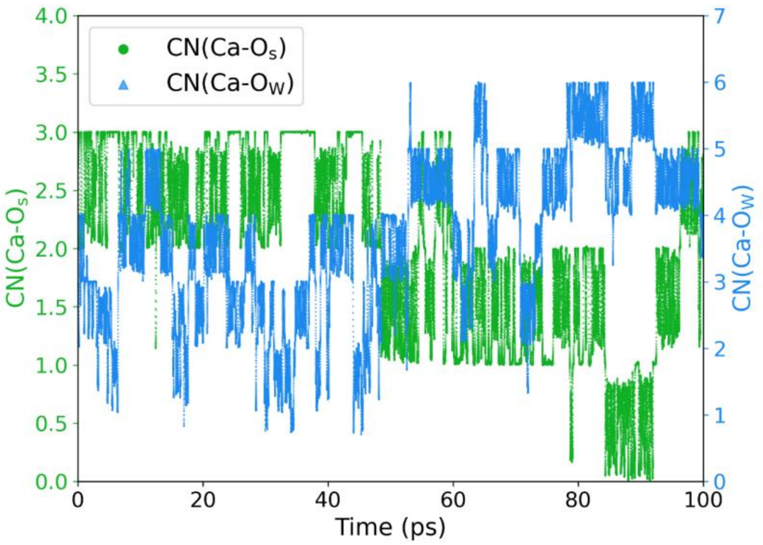

Supplementary Figure 2. Time evolution of CN(Ca-O$_s$) and CN(Ca-O$_w$) during the whole simulation time of the well-tempered metadynamics simulations for dissolution of Ca$_\alpha$.

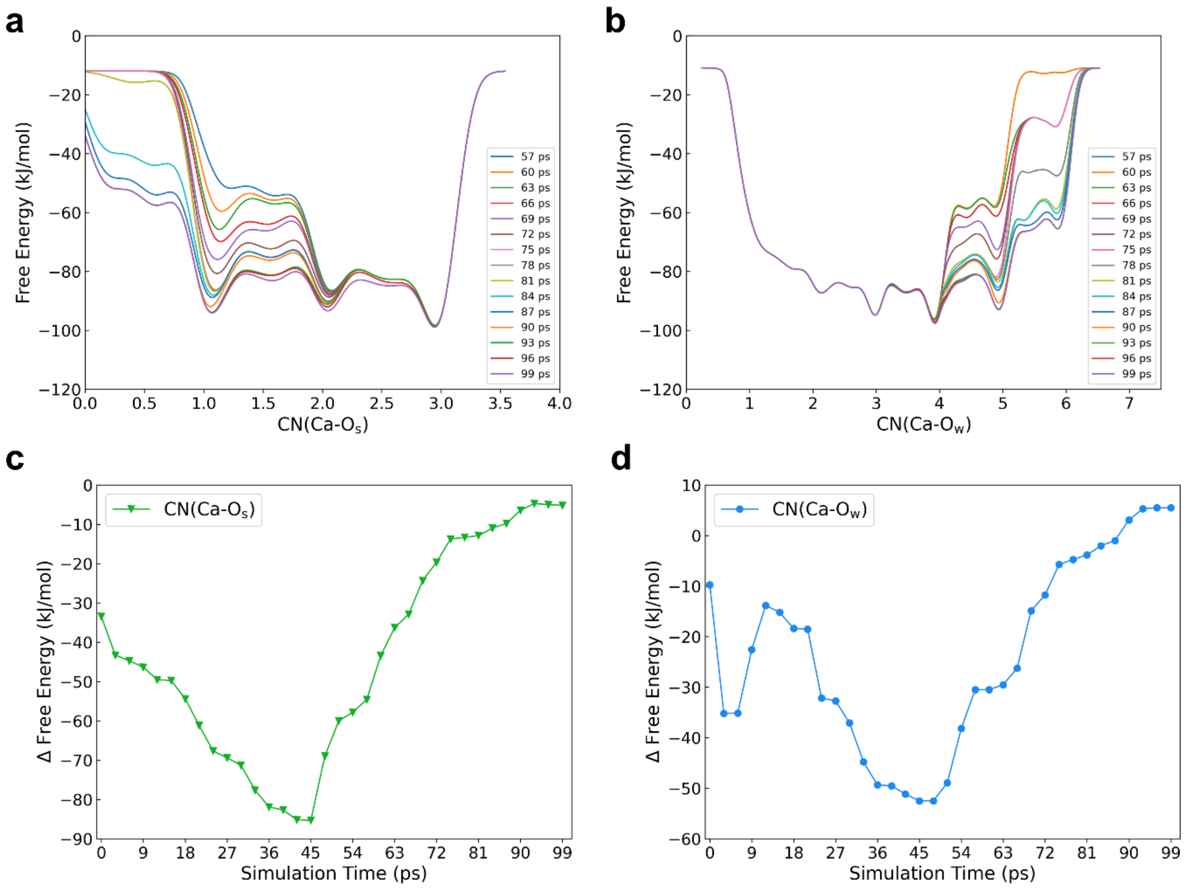

Supplementary Figure 3. Convergence tests for WT-MetaD simulations of dissolution of Ca_{α}. **a, b** The free energy surface as a function of the CN(Ca-O_s) or CN(Ca-O_W) every 3 ps (100 Gaussian kernels deposited) along the last 42 ps simulation time. **c, d** The free energy difference along CN(Ca-O_s) or CN(Ca-O_W) between two basins as a function of the whole simulation time.

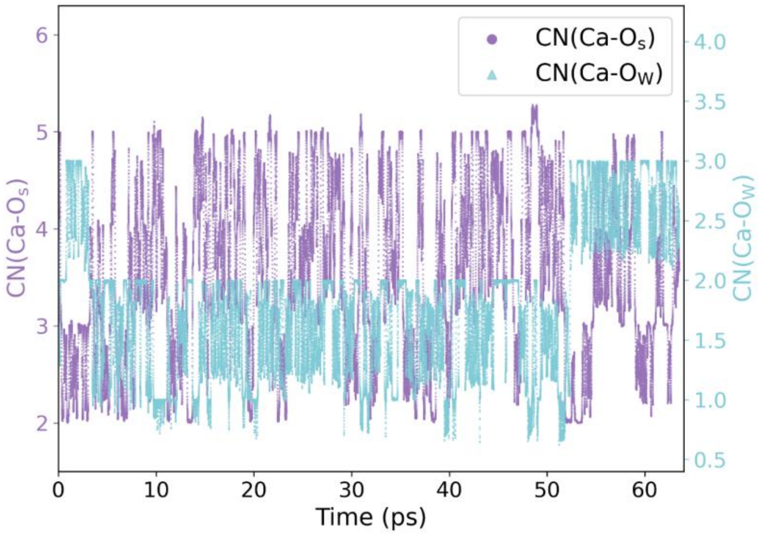

Supplementary Figure 4. Time evolution of CN(Ca-O_s) and CN(Ca-O_w) during the whole simulation time of the well-tempered metadynamics simulations for dissolution of Ca_β with CN(Ca-O_s) from 5 to 2.

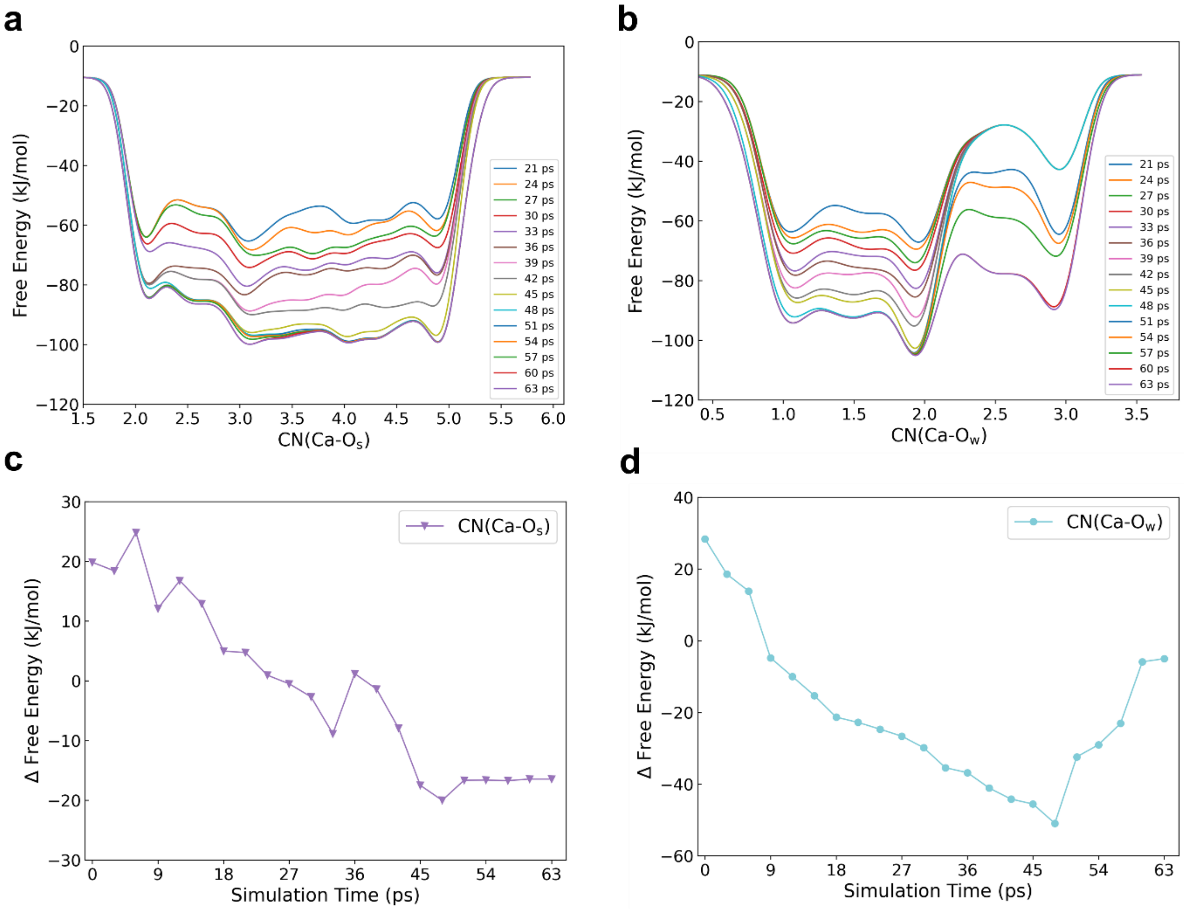

Supplementary Figure 5. Convergence tests for WT-MetaD simulations of dissolution of Ca_β with CN(Ca-O$_s$) from 5 to 2. **a, b** The free energy surface as a function of the CN(Ca-O$_s$) or CN(Ca-O$_w$) every 3 ps (100 Gaussian kernels deposited) along the last 42 ps simulation time. **c, d** The free energy difference along CN(Ca-O$_s$) or CN(Ca-O$_w$) between two basins as a function of the whole simulation time.

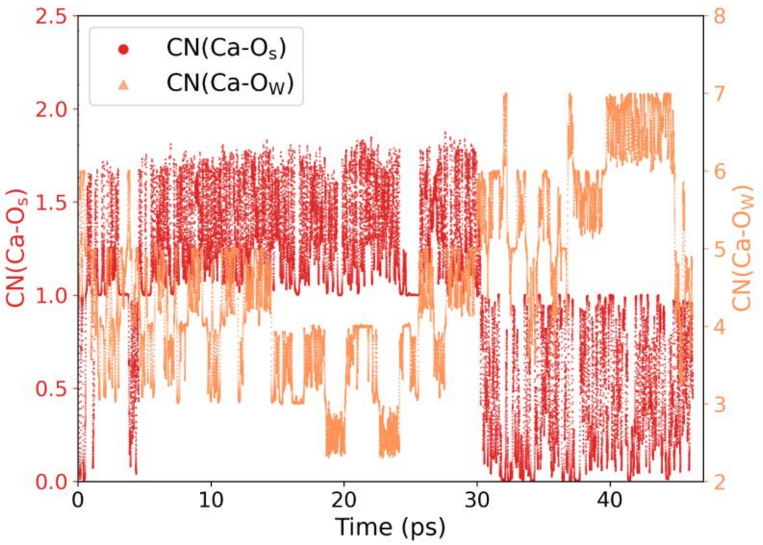

Supplementary Figure 6. Time evolution of CN(Ca-O_s) and CN(Ca-O_w) during the whole simulation time of the well-tempered metadynamics simulations for dissolution of Ca_β with CN(Ca-O_s) from 2 to 0.

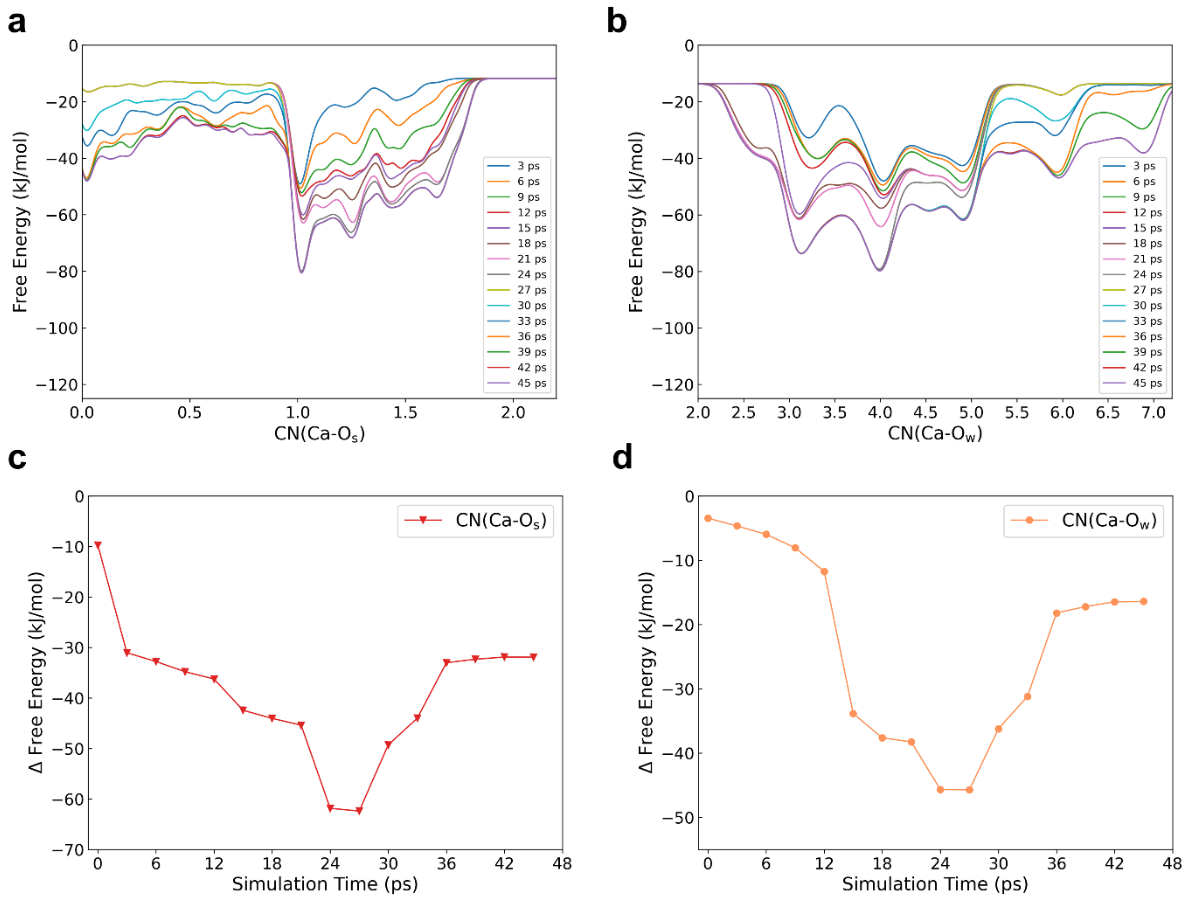

Supplementary Figure 7. Convergence tests for WT-MetaD simulations of dissolution of $Ca_β$ with CN(Ca-O_s) from 2 to 0. **a, b** The free energy surface as a function of the CN(Ca-O_s) or CN(Ca-O_W) every 3 ps (100 Gaussian kernels deposited) along the last 42 ps simulation time. **c, d** The free energy difference along CN(Ca-O_s) or CN(Ca-O_W) between two basins as a function of the whole simulation time.

Supplementary References

1. Kresse G, Furthmüller J. Efficiency of ab-initio total energy calculations for metals and semiconductors using a plane-wave basis set. *Computational materials science* **6**, 15-50 (1996).

2. Kresse G, Hafner J. Ab initio molecular dynamics for liquid metals. *Physical Review B* **47**, 558 (1993).

3. Perdew JP, Burke K, Ernzerhof M. Generalized gradient approximation made simple. *Physical review letters* **77**, 3865 (1996).

4. Durgun E, Manzano H, Pellenq R, Grossman JC. Understanding and controlling the reactivity of the calcium silicate phases from first principles. *Chemistry of Materials* **24**, 1262-1267 (2012).

5. Laanaiya M, Bouibes A, Zaoui A. Understanding why Alite is responsible of the main mechanical characteristics in Portland cement. *Cement and Concrete Research* **126**, 105916 (2019).

6. Qi C, Spagnoli D, Fourie A. DFT-D study of single water adsorption on low-index surfaces of calcium silicate phases in cement. *Applied Surface Science* **518**, 146255 (2020).

7. Kresse G, Furthmüller J. Efficient iterative schemes for ab initio total-energy calculations using a plane-wave basis set. *Physical review B* **54**, 11169 (1996).

8. Saritas K, Ataca C, Grossman JC. Predicting electronic structure in tricalcium silicate phases with impurities using first-principles. *The Journal of Physical Chemistry C* **119**, 5074-5079 (2015).

9. Monkhorst HJ, Pack JD. Special points for Brillouin-zone integrations. *Physical review B* **13**, 5188 (1976).

10. Noguera C. Polar oxide surfaces. *Journal of Physics: Condensed Matter* **12**, R367 (2000).

11. Tasker P. The stability of ionic crystal surfaces. *Journal of Physics C: Solid State Physics* **12**, 4977 (1979).

12. Li Q, García-Muelas R, López N. Microkinetics of alcohol reforming for H 2 production from a FAIR density functional theory database. *Nature communications* **9**, 1-8 (2018).

13. Garcı´a-Muelas R, Li Q, Lopez N. Density functional theory comparison of methanol decomposition and reverse reactions on metal surfaces. *ACS Catalysis* **5**, 1027-1036 (2015).
