# Supplementary material for: Ab initio mechanism revealing for tricalcium silicate dissolution
Source: Nat Commun. 2022 Mar 10;13:1253. doi: 10.1038/s41467-022-28932-2 (PMC8913775; doi:10.1038/s41467-022-28932-2)
Supplement: Supplementary file 3 — Description of Additional Supplementary Files [file 41467_2022_28932_MOESM3_ESM.docx]

**Description of Additional Supplementary Files**

**File Name: Supplementary Movie 1
Description:** an additional 30 ps equilibrium AIMD simulation for the water/Ca3SiO5 interface after the dissolution of the calcium ion.
